# How Do Patients with Hypermobile Ehlers-Danlos Syndrome Cope with This Medical Condition? An Analysis of Autobiographical Narratives in Relation to Pain Perception and Affect Regulation Capabilities

**DOI:** 10.3390/healthcare13060636

**Published:** 2025-03-14

**Authors:** Alessia Renzi, Claudia Celletti, Michela Di Trani, Marta A. S. Vizzini, Lorenzo Colaboni, Giada Petronelli, Massimo Pasquini, Filippo Camerota, Rachele Mariani

**Affiliations:** 1 Department of Dynamic and Clinical Psychology and Health Studies, Sapienza University of Rome, Via degli Apuli 1, 00185 Rome, Italy; alessia.renzi@uniroma1.it (A.R.); marta.vizzini@uniroma1.it (M.A.S.V.); lorenzo.colaboni@uniroma1.it (L.C.); rachele.mariani@uniroma1.it (R.M.); 2 Department of Life Sciences, Health, and Health Professions, Link Campus University, 00165 Rome, Italy; clacelletti@gmail.com; 3 Physical Medicine and Rehabilitation Division, Umberto I University Hospital, 00185 Rome, Italy; giadapetronelli@gmail.com (G.P.); filippo.camerota@uniroma1.it (F.C.); 4 Department of Human Neurosciences, Sapienza University of Rome, 00185 Rome, Italy; massimo.pasquini@uniroma1.it

**Keywords:** hypermobile Ehlers-Danlos syndrome, affect regulation, pain, autobiographical memories, referential process, linguistic characteristics

## Abstract

Background/Objectives: Hypermobile Ehlers-Danlos syndrome (hEDS) is the most common form of EDS, characterized by joint hypermobility, skin findings, and joint pains or recurrent dislocations that may also be associated with other several extra-articular symptoms. A deficit in the affect regulation represents a risk element in the development of both physical and mental health, as well as in a greater pain perception. The present study aims at exploring the associations between linguistic characteristics associated with different autobiographical memories and affect regulation and pain measures in patients affected by hEDS. A further aim is to explore the possible differences in linguistic measures between different episodes. Methods: Twenty-five patients with hEDS diagnoses (mean age = 38.32; SD = 17.00; 23 female) in treatment at the Physical Medicine and Rehabilitation Department of Umberto I Hospital in Rome completed a socio-demographic questionnaire, the Difficulties in Emotion Regulation Scale (DERS), the 20-item Toronto Alexithymia Scale (TAS-20), and the Brief Pain Inventory (BPI), as well as an interview aimed at collecting memories regarding neutral, positive, and negative events and the medical condition. The transcriptions of the interviews were analyzed using a computerized linguistic measure of the referential process (RP). Results: A correlational analysis showed several significant associations among the linguistic measures, affect regulation, and perception of pain, applied to neutral, positive, and disease condition narratives. Only few significant associations emerged regarding the negative episode. Moreover, significant differences emerged between the neutral event compared with the positive, negative, and diagnosis episodes, especially with the latter. Conclusions: The present findings seem to confirm the association between affect regulation, pain, and linguistic measures, sustaining an elaborative process. Specifically, the experience of chronic pain associated with the discovery of the rare disease becomes a meaningful experience in one’s life condition and supports the ability to cope with the experience of chronicity.

## 1. Introduction

Ehlers-Danlos syndromes (EDS) encompass a group of inherited connective tissue disorders, typically characterized by excessive joint mobility, unusually elastic skin, and fragile tissues [1]. The 2017 International Classification of EDS and related disorders outlines thirteen clinical subtypes, each distinguished by variations in key phenotypic traits, additional defining characteristics, and patterns of inheritance. Among these, hypermobile Ehlers-Danlos syndrome (hEDS) is the most prevalent [2], affecting roughly 1 in 5000 individuals, representing 80–90% of all cases [3]. As for hypermobility in general, also for hEDS, an overt excess in females has been observed (females = 6–57% vs. males = 2–35%) [4,5].

In addition to musculoskeletal complications, individuals with hEDS frequently experience a variety of symptoms affecting the cardiovascular, gastrointestinal, genitourinary, and nervous systems, further contributing to the chronic nature of the condition [6]. Individuals with EDS commonly experience a broad spectrum of symptoms, with pain reported by over 95% of patients. Non-specific central nervous system manifestations, such as headaches and dysautonomia, occur in nearly 90% of cases, while severe fatigue affects a similar proportion. Additionally, gastrointestinal complications, including acid reflux and gastroparesis, are observed in approximately 80% of individuals [7]. A major challenge in managing hEDS is the subjective nature of many of the reported symptoms, which often lack a clear organic cause. As a result, patients are frequently misclassified as “somatizers”, with physicians sometimes attributing their symptoms to psychological conditions before an accurate diagnosis is established [7]. However, research underscores the significant impact of psychological factors—such as fear, emotional distress, and negative emotions—on the clinical outcomes of individuals with hEDS. Chronic pain in patients with hEDS can severely affect mental well-being, increasing the risk of psychopathological symptoms and negative emotional states [8]. According to a recent systematic review [9], hEDS showed a high comorbidity with language disorders (63.2%), attention-deficit/hyperactivity disorder (ADHD) (52.4%), anxiety (51.2%), learning disabilities (42.4%), and depression (30.2%). Moreover, in individuals with hEDS [10], a notably higher prevalence of personality disorders has been identified compared with matched control groups. This implies that psychiatric symptoms in hEDS may not be solely reactive but could also stem from an underlying predisposition [11].

In summary, hEDS presents significant challenges, with a lengthy diagnostic process and a substantial impact on patients’ quality of life. Research highlights the need for greater attention to its psychological aspects to deepen understanding and support the development of targeted psychological interventions [9,12].

A valuable way to explore how individuals navigate emotionally challenging experiences, such as living with hEDS, is through language analysis. The way that they articulate their experiences can offer insight into their ability to connect emotions with words and thoughts [13]. Autobiographical memory involves the recall of personal experiences or episodes, playing a pivotal role in preserving self-continuity [14], allowing individuals to draw on past experiences to face new challenges and reserve an integrated sense of identity [15]. Bucci’s Multiple Code Theory (MCT) integrates both physical and mental dimensions, enabling the exploration of connections between different levels of information processing [16,17,18]. According to MCT, analyzing spoken or written language offers valuable insight into how various processing mechanisms interact and structure information [15,16,17,19,20,21]. These levels consist of: (a) the sub-symbolic system, responsible for processing non-verbal cues and shaping the emotional core; (b) the non-verbal symbolic system, which structures discrete images and representations derived from sub-symbolic processing; and (c) the verbal symbolic system, where these images and representations are translated into language. Life experiences are stored within dynamic memory schemas, integrating both symbolic and sub-symbolic elements. These schemas develop in early life, continue into adulthood, and evolve through new experiences. Likewise, emotion schemas encode patterns of bodily sensations and personal memory representations [22]. Emotion schemas organize visceral and emotional experiences, similar to how memory schemas structure autobiographical events, highlighting the interaction between memory, emotion, and perception. In MCT, three functions connect experience to language: arousal, which triggers an emotional schema; symbolizing, the process of associating an image with arousal; and reflection/reorganization, which allows for the restructuring of meanings to create a new significance [17]. These phases form the referential process (RP) that facilitates the sharing of emotional experiences with others and acts as a mechanism for emotional self-regulation [13,16,17,19,23,24]. Internal conflict or a traumatic experience, like suffering from a rare genetic syndrome, can disrupt or dysregulate the RP, leading to difficulties in emotional self-regulation, including challenges in expressing emotions verbally and forming a cognitive understanding of emotional experiences [15,25,26]. In this context, inadequate emotional regulation skills may pose a risk, as successfully managing such events often depends on staying in tune with one’s emotions and needs [27]. Indeed, the international literature sustains that the ability to regulate emotions, known as affect regulation, is a key factor in understanding the onset and evolution of symptoms across various medical conditions [20,28,29], representing a risk factor for both the development and severity of physical and mental health disorders [30]. Precisely, a broad literature on psycho-physical health underscores that individuals with physical or psychological disorders exhibit higher levels of affect dysregulation compared with healthy populations [27,31,32].

Within the field of affect regulation disorders and their connection to health and pathology, the concept of alexithymia has been extensively studied and debated, receiving substantial theoretical and empirical attention over the past decades [33]. Alexithymia is a complex construct involving difficulties in identifying and expressing emotions, a reduced capacity for imagination, and a preference for a concrete, externally oriented cognitive style [26,32]. Recent research redefines alexithymia as an emotion-dysregulation disorder, characterized by difficulties in managing distress through imagination, problem-solving, and expressing needs for social support [27,32]. Individuals with emotional regulation difficulties often report more somatic symptoms, especially in response to stress, negative situations, or perceived threats, as may be considered when suffering from hEDS [34,35]. As a result, individuals with high levels of alexithymia/ affect dysregulation often experience more intense clinical pain [36,37,38,39]. According to Lankes and colleagues [35], it is suggested that difficulties in affect regulation may exacerbate pain and negatively impact clinical outcomes. However, the relationship between affect regulation and pain remains debated, with conflicting findings requiring further investigation.

### Aims

This study explores RP measures in autobiographical narratives of patients with hEDS. Specifically, we compare linguistic patterns in narratives describing neutral, positive, negative, and illness-related events prompted by visual stimuli (see Measures section). By analyzing narratives with varying emotional content, we aim to identify linguistic features that are unique to the experience of living with hEDS. Additionally, we examine the associations between RP linguistic measures, affect regulation, and pain levels. Considering the absence of specific previous literature on this topic, only a general hypothesis can be assessed. In this light, our first hypothesis is to find diverse elaboration and symbolization competencies according to the specific memories expressed. Moreover, we hypothesized to find a different association between the linguistic characteristic of the different episodes and affect regulation and pain measures.

## 2. Materials and Methods

### 2.1. Participants

All participants were recruited from the outpatient clinic at the Division of Physical Medicine and Rehabilitation, Umberto I University Hospital, within the multidisciplinary service for Hereditary Connective Tissue Disorders. Participation in the study was offered based on the following inclusion criteria: (a) a confirmed diagnosis of hEDS according to the 2017 criteria; and (b) no diagnosed neurological conditions.

According to the inclusion criteria, 28 patients were invited to participate in the study, all of whom agreed. However, three patients with hEDS were excluded from the data analysis due to an incomplete study protocol completion. Consequently, the final sample consisted of 25 patients, with a mean age of 38.32 (SD = ± 17.00). Only two participants were male. A detailed overview of the socio-anamnestic data and psychological test scores is provided in Table 1 and Table 2.

### 2.2. Measures

Socio-anamnestic Questionnaire. A socio-anamnestic questionnaire was precisely developed to obtain information regarding age, gender, education level, social status, occupation, and information about the disease at the time of diagnosis.

Difficulties in Emotion Regulation Scale (DERS) [40,41]. It is a 36-item self-report questionnaire aimed at evaluating difficulties in emotion regulation, especially those regarding negative emotions. The instrument includes six subscales: Non-acceptance of Negative Emotional Responses (6 items), Difficulties Engaging in Goal-Directed Behavior When Distressed (5 items), Difficulties Controlling Impulsive Behaviors When Distressed (6 items), Lack of Emotional Awareness (6 items), Limited Access to Effective Emotion Regulation Strategies (8 items), and Lack of Emotional Clarity (5 items). The DERS provides both a total score and subscale scores, with higher scores indicating greater difficulties in emotion regulation. The DERS has demonstrated robust psychometric properties across both clinical and nonclinical populations [42,43,44].

20-item Toronto Alexithymia Scale (TAS-20) [45,46]. It is the most widely used self-report measure to evaluate alexithymia, consisting of 20 items organized into three sub-scales: difficulty identifying feelings (DIF), difficulty describing feelings (DDF), and externally oriented thinking (EOT). The TAS-20 provides a total score as well as scores for the three subscales, with higher scores showing a higher alexithymia level. The TAS-20 has demonstrated satisfactory psychometric properties, including internal consistency and a strong test–retest reliability.

Brief Pain Inventory (BPI) [47,48]. It is a self-report measure evaluating the severity of pain and the related impact on various aspects of a patient’s daily life through an 11-point numerical rating scale. The BPI assess both the intensity of pain (sensory dimension) and the interference of pain in the patient’s life (reactive dimension), which are both activity and affective sub-dimensions [49]. The scores range from 0 (no pain) to 10 (pain as severe as you can imagine). The BPI has demonstrated satisfactory psychometric properties, including internal consistency and a strong test–retest reliability.

Semi-structured interview: A specific semi-structured interview to gather autobiographical narratives was conducted. The interview included three visual stimuli from the International Affective Pictures System battery—IAPS [50]. This battery contains pictures and photographs of people, animals, objects, and landscapes capable of provoking both positive and negative emotions. Specifically, the slides are labelled with standardized valence values, so that the stimuli were classified as negative, neutral, or positive in emotional content. For this study, a selection of three images was selected according to the standardized values assigned: a book (neutral affect), a seaside (positive affect), and a shark (negative affect). This battery has been validated in several countries, supporting IAPS as a good technical instrument for scientific studies [51]. For each image, the interviewer posed the following question: “I will now show you an image. Can you tell me a memory or episode from your life that comes to mind?” After the three visual stimuli, the interviewer asked about the participant’s experience with their disease condition by posing the following question: “Can you tell me about a critical event related to your medical condition?”

Computerized linguistic measures of the referential process: The Italian Discourse Attribute Analysis Program (IDAAP) [52] has been employed to investigate the transcribed text and analyze the referential process (RP). IDAAP has been developed by Maskit [53] and applies both weighted and unweighted dictionaries, generating results through an exponential smoothing algorithm. For this study, we selected specific dictionaries and measures that were specifically constructed and validated for the English and Italian languages to assess the RP in written narratives [54].

- The Italian Weighted Referential Activity Dictionary (IWRAD): The Italian Weighted Referential Activity Dictionary (IWRAD) is a computerized measure of the symbolizing phase [55] in the Italian language. It has been considered a direct measure of the symbolizing process. It contains a list of 9596 frequently used Italian words, each assigned a weight between 0 and 1, with 0.5 as the neutral value. Higher scores show a higher level of symbolizing, highlighting a higher level of concreteness, specificity, clarity, and imagery in the speech. Moreover, high scores on this index represent a high integration of emotional schemas as understood through MCT.
- The High WRAD Proportion (HPIWRAD) and the Mean High WRAD (HIWRAD) are two sub-indexes of the WRAD, indicating indirect measures of the symbolizing phase.
- Italian Weighted Reflection and Reorganization List (IWRRL): The Italian Weighted Reflection and Reorganization List (IWRRL) [56] contains a series of Italian weighted words regarding the reorganization and reflection phase. It is not about abstract reflection, but rather, a person’s reasoning related to an experience that has been vividly experienced. This measure represents a direct measure of the reflection/reorganizing phase and higher scores correspond to higher reflection/reorganization competences. The IWRRL is a list of Italian weighted words, ranging from 0 and 1, with 0.5 as a neutral value, referring to the reorganization and reflection function. The list consists of 1633 words with coverage of 89% of the Italian language as it normally spoken [56]. High scores on this measure represent high reflection/reorganizing as understood through MCT.
- The High WRRL Proportion (HPIWRRL) and the Mean High WRRL (HIWRRL) are two sub-indexes of the WRRL, indicating indirect measures of the reflection and reorganization phase.
- The Italian Reflection Dictionary (IRefD) [55] is a specific dictionary comprising Italian words associated with cognitive and logical processes, as well as communication needing cognitive functions. It measures abstract reflection and emotional distancing through the proportion of IRefD terms in language. Studies on psychotherapy [16,57] have shown it to be a reliable indicator of defensive intellectualization.
- The covariation between REF and WRAD evaluates the extent to which the two (smoothed) measures are simultaneously high or low. It has been shown to be related to clinical judgments of effectiveness since it is a further index of the symbolization phase [55].
- The Italian Sensory Somatic Dictionary (ISensD) comprises a series of Italian words related to the body and bodily activities, and to sensory processes and/or descriptions of symptoms [58]. The number of ISensD words in language is a measure of the arousal of the bodily, subsymbolic aspects of emotion schemas.
- The Italian Affect Dictionary (AffD): Designed to assess emotional expression, the AffD categorizes affect into negative (IAffN), positive (IAffP), and neutral (IAffZ) components [59].
- The Italian Disfluency Dictionary (IDFD): The Italian DisFluency Dictionary (IDFD) is a small set of words (11 words), as well as repeated words, incomplete words, and filled pauses that people tend to use when struggling to communicate [60]. A score on this index corresponds to the proportion of IDFD words present in the speech. High scores typically characterize the arousal phase in which emotion schemas are activating.

### 2.3. Procedure

The research was conducted in compliance with the code of ethics of the World Medical Association (Declaration of Helsinki) for experiments involving human participants. Ethical approval for the present study was granted by the Hospital Ethical Committee. The data collection for the present study was realized in a period of three months. During a routine visit at the medical department, the physician screened the patients with hEDS for eligibility. Subsequently eligible patients were approached by the psychologist, who asked for their consent to participate in the study. Each participant signed an informed consent before proceeding to complete the measures and the interview. The entire study protocol was realized within the medical center by a qualified psychologist in a dedicated room, safeguarding the participants’ privacy. Each interview was recorded and transcribed, and the transcribed text for each topic was separately analyzed using the computerized IDAAP system.

### 2.4. Statistical Analysis

Data analyses for the present study were executed through the Statistical Package for Social Science—24 (SPSS version 24, Armonk, NY, USA). Continuous variables are presented as means and standard deviations, while categorical variables are expressed as frequencies and percentages. To analyze the differences in RP linguistic indices across various narrative episodes (neutral episode versus all other episodes), a paired-sample Student’s *t*-test was conducted. A Pearson’s correlation analysis was used to examine the relationships between RP linguistic measures, affect regulation, and pain scores. Additionally, correlations with socio-anamnestic factors (such as age and time since diagnosis) were explored specifically for RP indices related to medical condition narratives. A *p* value < 0.05 was considered significant.

## 3. Results

Table 3 reports the differences between the RP linguistic measures applied to different autobiographical episodes. Several significant differences comparing neutral episodes with positive and negative episodes, and the illness condition, respectively, emerged (see Table 3). We encourage caution in considering the significances that emerged since the reduced sample size.

A correlation analysis showed several significant associations between the RP linguistic measures applied, respectively, to neutral, positive, and illness episodes and affect regulations and pain measures (see Table 4, Table 5 and Table 6). Regarding the RP linguistic measure applied to the negative episode, only few significant associations with pain and affect regulation dimensions emerged. Specifically, TAS-20 difficulties in identifying feelings showed a negative association with the DisFluency index (r = −0.397; *p* < 0.05), the Covariate IRef_IWRAD index reported a positive association with the DERS awareness scale (r = 0.416; *p* < 0.05), and a negative one with BPI pain intensity (r = −0.445; *p* < 0.05). Lastly, the Covariate IWRAD_IWRRL index showed a negative association with TAS-20 difficulties in describing feelings (r = −0.439; *p* < 0.05).

Moreover, regarding the associations between the RP linguistic measure applied to the illness episode with socio-anamnestic variables, only a significant negative association between the time since diagnosis and the senso-somatic index was found (see Table 6).

## 4. Discussion

### 4.1. Discussion on the Differences in the RP Measures Applied to Neutral, Positive, Negative, and Illness Autobiographical Memories

The aim of our study is to examine the RP measures applied to narratives of autobiographical memories in individuals with hEDS. To our knowledge, this is the first study exploring the relationship between narrative patterns, emotional elaboration, and pain perception in this clinical population. Our primary exploratory hypothesis was to compare the linguistic patterns in autobiographical narratives of emotionally neutral events with those describing positive, negative, and illness-related experiences, elicited through visual stimuli using a structured interview protocol. By analyzing these narratives, we aimed to investigate the activation of emotional schemas using the multiple code theory that may contribute to pain processing and the elaboration of a challenging diagnosis. The comparison between neutral episodes, positive/negative stimuli, and critical moments of illness revealed significant differences. There were no significant differences between the neutral, positive, and negative episodes, except for the illness-related condition; regarding this, the word count was significantly higher in the latter. This suggests that the experience of illness elicits a more elaborate narrative. Regarding affective dictionaries, the results align with the presented stimuli. Specifically, negative emotions (IAffN) were significantly higher in the neutral episode than in the positive episode, but significantly lower than in both the negative episode and the illness-related condition. Conversely, positive emotions (IAffP) were significantly lower in the neutral episode than in the positive episode, but higher than in the negative episode and the illness-related condition. When considering the total emotional content (IAffS), the positive episode contained the highest number of affective words. The positive stimulus appeared to encourage a more coherent and less ambivalent narrative, fostering greater engagement with the experience and enhancing the ability to symbolize and reflect (covariate IWRAD_IWRRL). In other words, the positive stimulus facilitated a more open engagement with one’s experiences. The direct indices of RP (i.e., IWRAD and IWRRL) did not show significant differences when comparing the autobiographical episodes; however, significant variations emerged through the indirect indices. Specifically, the illness-related episode demonstrated a greater activation of the referential cycle, with increased engagement in the symbolization phase (significantly more negative IREF_IWRAD covariate index) and the reflection/reorganization phase (significantly higher HPWRRL) compared with the neutral episode. This suggests that while positive stimuli elicit simpler, more accessible narratives, illness-related episodes provoke deeper emotional processing and re-elaboration. These results are strongly connected to the multiple code theory and the relevance of subsymbolic activation thanks to the imagine stimulus. In fact, Negri and Ongis [61] demonstrated a better form definition in the imagine definition, including color definition, and with fewer silhouettes of people, eliciting responses that were higher in the symbolizing phase, and also higher in the degree to which the two measures varied together. In other words, the stimulus is clearer and the episode more is the RP.

### 4.2. Discussion on the Associations Between RP Measures and Affect Regulation and Pain in Different Autobiographical Memories

A secondary objective of our study was to examine the relationship between linguistic measures elicited by different stimuli and both disease-related pain perception and emotional regulation abilities. A linguistic analysis of the neutral stimulus revealed that abstract words (IRefD) positively correlated with DERS difficulties in impulse control, emotional regulation strategies, and alexithymic traits, particularly those related to difficulties in identifying emotions. Additionally, higher abstract word (IRefD) scores were associated with greater pain interference in daily activities. Sensory-somatic words (ISenS) were positively correlated with both pain intensity and its interference in daily life, while showing a negative correlation with TAS-20 Externally Oriented Thinking, an alexithymia factor.

Regarding symbolization in narrative processes (both IWRAD and Covariate IRef_IWRAD), stronger symbolic processing in the narrative stimulus was linked to lower emotional dysregulation and a reduced alexithymic characteristic regarding difficulties in identifying and describing feelings. Interestingly, a greater narrative capacity for the reflection and reorganization of autobiographical experiences correlated with higher perceptions of pain and disability. These findings suggest meaningful connections between emotional regulation difficulties, pain perception, and linguistic patterns in neutral episodes, aligning with the multiple code theory framework and emotional schema activation. Notably, the correlations observed in the positive stimulus condition were less pronounced compared with the neutral stimulus, further reinforcing the distinction in narrative engagement across different emotional contexts.

The relationship between emotional schema activation and pain became more evident as emotional arousal (IDF) was negatively correlated with pain perception and alexithymia. The link between symbolization (both IWRAD and Covariated IREF_IWRAD) and pain perception was strengthened, showing that greater symbolization correlated with heightened pain perception. Similarly, a higher reflection and reorganization index (IWRRL) was associated with increased pain perception and reduced alexithymia. These findings suggest that while positive emotional arousal reduces pain perception, greater abilities to symbolize and reflect on emotions enhance the awareness of physical pain. This result opens up important reflections on the use of emotional activations and on the attention bias of positive stimuli [62], where emotional activation can elicit a defensive effect on the one hand and an exploratory opening on the other. More studies should be carried out on the relationship between positive stimuli and pain perception. Few correlations emerged between RP linguistic measures and the negative stimulus. However, emotional arousal (IDF) again showed a negative correlation with alexithymia. Notably, symbolic processes (Covariate IREF_IWRAD) in response to the negative stimulus reversed the trend, displaying a positive association with emotional dysregulation and a negative correlation with BPI pain intensity. This suggests that focusing on negative stimuli catalyzes symbolic processes. This result is particularly original with respect to the study of chronic pain conditions. In psychopathology-oriented studies, there is an inverse process, i.e., narration produces relief in the person’s experience. However, in this study, we speak of perceived physical pain, an element that is not evaluated in psychopathology. Here, a clinical vignette to point out an interesting link between the symbolizing process and pain perception:

“First episode that comes to mind is about XXXX when I was in a wheelchair all of a sudden and it wasn’t the best… especially because I like to get around, I like to do things, I’m a person who likes to be active, I don’t like to stay on the couch and do nothing all day long [...] it’s not that my legs didn’t work, my leg hurt and I couldn’t stand, I couldn’t lie down, I couldn’t stay in any position. It was two years of...banging around because the drugs weren’t working, things I couldn’t do...it ended up that I didn’t go out, I didn’t do anything [...] the pain stayed there for two years because the anti-inflammatories didn’t work, the painkillers didn’t work, the opiates didn’t work, in short, a bit of everything, then they gave me an infiltration but that didn’t work either, so a couple of months, at least, it must have been that I said enough, I have to stand up, I don’t feel like sitting anymore (laughs) here we have to do something the pain was actually maybe that too, to walk on it and say who cares… that a little bit compared to getting used to it, because obviously a little bit is me getting used to it in terms of pain.”

Finally, in autobiographical narratives of the critical illness-related episode, the presence of affective words, particularly positive ones (IAffP), was linked to emotional dysregulation, alexithymia, and pain perception. Additionally, the symbolization (both IWRAD and Covariate IREF_IWRAD) and reflection/reorganization indices (IWRRL) correlated positively with both emotional dysregulation and pain perception, reinforcing the intricate relationship between emotional processing, narrative elaboration, and pain experience.

### 4.3. Limitations

The present findings should be interpreted considering some limitations. Firstly, the small sample size, which may be partially due to the specific clinical population since hEDS has a very low prevalence. A second limitation is the low participation of male patients, which is due to the lower representation of this clinical condition in the male sex. Nevertheless, future studies should include a larger number of participants, particularly males, to explore the specific characteristics of this specific population and the potential differences in the elaborative process related to the syndrome compared with females. Additionally, it is important to note that recruitment was conducted at a single medical department, which may introduce potential selection bias. To overcome this limitation, future studies should enroll participants from multiple medical centers. A further limit is the use of self-report measures that may introduce social desirability bias. Future studies utilizing clinician-reported measures, such as semi-structured interviews for the assessment of alexithymia, may provide valuable insights. A final limitation is the absence of a control group, which should be included in future research to explore differences both in the general population and in individuals with other medical conditions.

## 5. Conclusions

The results of this study aim to establish a connection between autobiographical narration, emotional regulation processes, and the experience of pain in patients with hEDS. They highlight how autobiographical narration is closely linked to body perception and pain experience, both as a limiting factor in daily activities and as a psychological challenge to endure. The findings obtained through various emotional activation stimuli, combined with personal illness narratives, emphasize the significance of storytelling in activating and regulating emotional experiences. Our results are in line with many studies that show that increasing verbal competence and increasing the construction of stories about one’s life experience increase affective complexity [63]. In these patients, the processes of symbolization and the experience of reorganization are deeply intertwined with perceived pain and disability. The interview suggests that free narrative processes (neutral stimuli) can facilitate the expression of emotional patterns. On the other hand, critical episodes related to the disease activate levels of RP cycles that are, however, related to manifestations of emotional directivity and increased pain perception. This extremely interesting result highlights the importance of storytelling and the complexity of life experience. However, it should be emphasized that in the telling of the story, patients with hEDS do not activate a disconnected emotional pattern, as was found in women who told of their experience of medically assisted procreation [15,26], but the results show that the symbolic telling of their discovery of the diagnosis increases their more conscious contact with the experience of pain and the need to live with it and with disability, increasing their emotional complexity.

The study’s implications underscore the crucial role of psychological support in managing chronic illness, offering essential tools for pain regulation and emotional well-being. Psychological interventions aimed at enhancing emotional competencies appear crucial in improving pain management and psycho-physical health [64], as well as facilitating the reorganization of one’s experience following the diagnosis of such an invasive medical condition. In this context, psychological support can also help to reframe the pain experience, allowing individuals to integrate it into the broader narrative of their life journey. Moreover, collaboration between physicians and psychologists appears desirable within a bio-psycho-social model of disease and care, ensuring that all the relevant dimensions are addressed and fostering a joint effort to enhance patients’ quality of life.

## Figures and Tables

**Table 1:** Participants’ socio-anamnestic characteristics.

Variables	M/n.	SD/%
Age	38.32	17.00
Time since diagnosis	9.08	6.16
Employment status		
Unemployed	4	16
Employed	15	60
Student	6	24
Educational Qualification		
Middle School Diploma	3	12
High School Diploma	15	60
Bachelor’s degree	5	20
Postgraduate degree	2	8
Marital status		
Single	14	56
Cohabiting	3	12
Married	7	28
Separated	1	4
Children		
Yes	14	56
No	11	44

**Table 2:** Participants’ psychophysical measures investigated.

Variables	M	SD	Minumum Obtained	Maximum Obtained/Possibile
Difficulties in Emotion Regulation Scale				
Total score	92.24	36.80	40	173/180
Non Acceptance	16.84	8.00	7	30/30
Goals	14.80	5.88	6	25/25
Impulse	14.92	8.14	6	29/30
Awareness	15.12	6.42	6	28/30
Strategies	18.48	9.11	9	38/40
Clarity	12.08	5.49	5	23/25
20-item Toronto Alexithymia Scale				
Total score	53.36	12.92	25	76/100
DDF Difficulty Describing Feelings	21.28	8.03	9	25/25
DIF Difficulty Identifing Feelings	15.12	4.72	7	35/35
EOT Externally Oriented Thinking	16.96	4.85	8	26/40
Brief Pain Inventory				
Pain Intensity	5.18	2.06	1	7.75/10
Pain Interference	4.61	2.32	0.71	8.57/10

**Table 3:** Significant differences in the linguistic measures between the narrative regarding neutral episodes and other episodes.

Linguistic Measures	Neutral Episode		Positive Episode				Negative Episode				Disease Condition			
	M	SD	M	SD	*t*	*p*	M	SD	*t*	*p*	M	SD	*t*	*p*
Words	383.32	280.983	393.68	433.448	−0.1721	0.86	449.20	337.280	−1.337	0.19	1075.44	867.398	−4.1439	0.01 **
IDFD	0.0773	0.0376	0.0768	0.0387	0.0716	0.94	0.0791	0.0356	−0.312	0.75	0.0689	0.0408	1.401	0.17
IAffN	0.0112	0.0077	0.0065	0.0066	2.0391	0.05 *	0.0236	0.0153	−3.927	0.01 **	0.0161	0.0073	−2.806	0.01 **
IAffP	0.0268	0.0155	0.0423	0.0261	−2.8022	0.01 **	0.0161	0.0128	2.656	0.01 **	0.0165	0.0145	2.565	0.01 **
IAffS	0.0426	0.0193	0.0551	0.0252	−2.158	0.04 *	0.0433	0.0172	−0.172	0.86	0.0371	0.0173	1.069	0.29
IAffZ	0.0045	0.0040	0.0062	0.0062	−1.3377	0.19	0.0035	0.0047	0.866	0.39	0.0044	0.0029	0.166	0.86
IRefD	0.0357	0.0188	0.0289	0.0092	1.5922	0.12	0.0273	0.0147	2.627	0.01 **	0.0314	0.0185	1.555	0.13
IsensD	0.0540	0.0332	0.0582	0.0238	−0.5270	0.60	0.0588	0.0212	−0.582	0.56	0.0486	0.0157	0.775	0.44
IWRAD	0.4988	0.0055	0.5038	0.0044	−3.5357	0.01 **	0.5011	0.0060	−1.471	0.15	0.4994	0.0054	−0.385	0.70
IWRRL	0.5436	0.0058	0.5430	0.0060	0.4274	0.67	0.5437	0.0046	−0.037	0.97	0.5459	0.0050	−1.820	0.08
HIWRAD	0.0098	0.0034	0.0104	0.0042	−0.6037	0.55	0.0087	0.0036	0.832	0.42	0.0089	0.0062	0.418	0.68
HPIWRAD	0.4485	0.2034	0.6250	0.1690	−2.6125	0.02 *	0.5774	0.1989	−2.774	0.01 **	0.4656	0.2258	−0.233	0.82
HIWRRL	0.0447	0.0057	0.0438	0.0073	0.3753	0.71	0.045	0.0048	−0.156	0.87	0.0457	0.0035	−0.734	0.47
HPIWRRL	0.9848	0.0225	0.9914	0.0085	−1.3980	0.18	0.9944	0.0072	−1.833	0.09	0.9969	0.0049	−2.088	0.05 *
Cov IRef_IWRAD	−0.0576	0.2698	−0.0365	0.2628	−0.2611	0.79	−0.1041	0.2695	0.591	0.56	−0.2103	0.2704	2.226	0.03 *
Cov IWRAD_IWRRL	−0.0420	0.2980	0.2859	0.2321	−4.6441	0.01 **	0.1239	0.3215	−2.775	0.01 **	−0.004	0.3918	−0.450	0.65

* *p* < 0.05; ** *p* < 0.01. Legend: Words = number of words; IDFD = Italian DisFluency Dictionary; IAffN = Negative Affects; IAffP = Positive Affects; IAffS = Sum of Affects; IAffZ = Neutral Affects; IRefD = Italian Reflection Dictionary; ISensD = Italian Sensory Somatic Dictionary; IWRAD = Italian Weighted Referential Activity Dictionary; HIWRAD = Mean High—Italian Weighted Referential Activity Dictionary; IWRRL = Italian Weighted Reflection and Reorganization List; HPIWRAD = High WRAD Proportion; HIWRRL = Mean High—Italian Weighted Reflection and Reorganization List; HPIWRRL = High WRRL Proportion; Cov = covariation.

**Table 4:** Associations between affect regulation dimensions, pain, and neutral episode linguistic measures.

	Words	IDFD	IAffN	IAffP	IAffS	IAffZ	IRefD	ISenSD	IWRAD	IWRRL	Cov IRef_IWRAD	Cov IWRAD_IWRRL
DERS Total	−0.251	−0.163	0.176	0.248	0.248	−0.107	0.362	−0.116	−0.378	0.316	0.275	−0.285
DERS Non Accept	−0.136	0.031	0.135	0.241	0.276	0.132	0.343	−0.138	−0.429 *	0.336	0.382	−0.329
DERS Goals	−0.336	−0.268	0.197	0.077	0.082	−0.285	0.243	0.080	−0.434 *	0.320	0.189	−0.288
DERS Impulse	−0.332	−0.369	0.065	0.271	0.211	−0.160	0.465 *	−0.209	−0.041	0.360	0.319	−0.105
DERS Awareness	−0.157	0.171	0.092	0.107	0.096	−0.128	0.008	−0.231	−0.203	−0.194	0.116	−0.158
DERS Strategies	−0.199	−0.216	0.246	0.265	0.292	−0.096	0.410 *	−0.071	−0.384	0.374	0.112	−0.329
DERS Clarity	−0.115	−0.144	0.161	0.259	0.262	−0.054	0.288	0.034	−0.420 *	0.279	0.289	−0.236
TAS-20 Total	−0.131	−0.043	0.208	0.089	0.186	0.146	0.209	−0.334	−0.343	0.009	0.269	−0.297
TAS-20 DIF	−0.140	−0.199	0.074	0.177	0.172	0.000	0.474 *	−0.177	−0.248	0.127	0.404 *	−0.198
TAS-20 DDF	−0.296	0.020	0.193	−0.056	0.033	−0.001	0.004	−0.180	−0.530 **	0.028	−0.021	−0.368
TAS-20 EOT	0.172	0.196	0.243	−0.002	0.178	0.390	−0.233	−0.419 *	−0.024	−0.193	0.068	−0.104
BPI Intensity	0.307	−0.103	−0.181	−0.214	−0.203	0.200	0.237	0.447 *	−0.305	0.525 *	−0.106	−0.287
BPI Interference	−0.119	−0.149	−0.228	−0.032	−0.110	0.034	0.421 *	0.425 *	−0.385	0.658 **	−0.259	−0.445 *

* *p* < 0.05; ** *p* < 0.01. Legend: DERS = Difficulties in Emotion Regulation Scale; TAS-20 = Toronto Alexithymia Scale; TAS-20 DIF = Difficulties in Identifying Feelings; TAS-20 DDF = Difficulties in Describing Feelings; TAS-20 EOT = Externally Oriented Thinking; BPI = Brain Pain Inventory; Words = number of words; IDFD = Italian DisFluency Dictionary; IAffN = Negative Affects; IAffP = Positive Affects; IAffS = Sum of Affects; IAffZ = Neutral Affects; IRefD = Italian Reflection Dictionary; ISensD = Italian Sensory Somatic Dictionary; IWRAD = Italian Weighted Referential Activity Dictionary; IWRRL = Italian Weighted Reflection and Reorganization List; Cov = covariation.

**Table 5:** Associations between affect regulation dimensions, pain, and positive episode linguistic measures.

	Words	IDFD	IAffN	IAffP	IAffS	IAffZ	IRefD	ISenSD	IWRAD	IWRRL	Cov IRef_IWRAD	Cov IWRAD_IWRRL
DERS Total	−0.303	−0.239	−0.243	0.292	0.230	−0.034	0.044	0.063	−0.068	−0.329	−0.250	−0.001
DERS Non Accept	−0.248	−0.145	−0.140	0.283	0.318	0.252	−0.024	0.241	0.133	−0.285	−0.209	0.173
DERS Goals	−0.383	−0.109	−0.276	0.401 *	0.292	−0.201	0.043	0.053	−0.250	−0.331	−0.293	−0.150
DERS Impulse	−0.259	−0.320	−0.159	0.147	0.134	0.093	−0.034	0.186	0.066	−0.237	−0.299	0.097
DERS Awareness	−0.138	0.000	−0.179	0.002	−0.162	−0.468 *	0.073	−0.359	−0.343	−0.351	−0.122	−0.178
DERS Strategies	−0.282	−0.331	−0.187	0.272	0.245	0.052	0.057	0.074	0.012	−0.277	−0.169	0.000
DERS Clarity	−0.248	−0.251	−0.371	0.444 *	0.347	−0.056	0.155	0.037	−0.098	−0.212	−0.191	−0.034
TAS-20 Total	−0.224	−0.274	−0.115	0.116	0.120	0.119	0.158	0.110	−0.050	−0.313	−0.042	−0.021
TAS-20 DIF	−0.282	−0.439 *	−0.126	0.216	0.243	0.211	0.209	0.326	0.260	−0.187	−0.221	0.207
TAS-20 DDF	−0.261	−0.062	−0.159	0.215	0.131	−0.199	−0.004	−0.189	−0.353	−0.404 *	−0.123	−0.282
TAS-20 EOT	0.124	0.058	0.059	−0.258	−0.211	0.162	0.078	−0.063	−0.219	−0.130	0.373	−0.122
BPI Intensity	0.214	−0.413 *	0.014	0.093	0.171	0.289	−0.093	0.471 *	0.473 *	0.480 *	−0.105	0.339
BPI Interference	−0.187	−0.516 **	0.126	0.144	0.267	0.340	−0.068	0.275	0.541 **	0.237	−0.098	0.256

* *p* < 0.05; ** *p* < 0.01. Legend: DERS = Difficulties in Emotion Regulation Scale; TAS-20 = Toronto Alexithymia Scale; TAS-20 DIF = Difficulties in Identifying Feelings; TAS-20 DDF = Difficulties in Describing Feelings; TAS-20 EOT = Externally Oriented Thinking; BPI = Brain Pain Inventory; Words = number of words; IDFD = Italian DisFluency Dictionary; IAffN = Negative Affects; IAffP = Positive Affects; IAffS = Sum of Affects; IAffZ = Neutral Affects; IRefD = Italian Reflection Dictionary; ISensD = Italian Sensory Somatic Dictionary; IWRAD = Italian Weighted Referential Activity Dictionary; IWRRL = Italian Weighted Reflection and Reorganization List; Cov = covariation.

**Table 6:** Associations between affect regulation dimensions, pain, and illness episode linguistic measures.

	Words	IDF	IAffN	IAffP	IAffS	IAffZ	IRef	ISenS	IWRAD	IWRRL	Cov IRef_IWRAD	Cov IWRAD_IWRRL
Age	0.127	0.293	−0.047	0.009	−0.074	−0.358	−0.208	−0.134	−0.137	0.070	0.013	−0.167
Time Since Diagnosis	0.234	−0.082	−0.116	−0.175	−0.220	−0.109	−0.239	−0.450 *	0.104	−0.112	−0.010	0.043
DERS Total	−0.072	−0.038	−0.031	0.263	0.226	0.110	0.334	0.291	−0.305	0.452 *	−0.175	−0.222
DERS Non Accept	−0.075	0.066	−0.081	0.406 *	0.314	0.050	0.404 *	0.175	−0.486 *	0.350	−0.199	−0.386
DERS Goals	−0.130	0.057	0.074	0.035	0.094	0.194	0.261	0.429 *	−0.102	0.356	−0.199	−0.058
DERS Impulse	−0.049	−0.228	0.061	0.418 *	0.380	0.026	0.263	0.325	−0.282	0.323	−0.075	−0.187
DERS Awareness	0.189	0.143	−0.095	−0.098	−0.090	0.185	0.094	0.013	−0.130	0.409 *	0.156	−0.122
DERS Strategies	−0.179	−0.120	−0.039	0.282	0.231	0.062	0.288	0.323	−0.242	0.421 *	−0.288	−0.145
DERS Clarity	−0.085	−0.045	−0.081	0.158	0.115	0.097	0.392	0.202	−0.256	0.477 *	−0.261	−0.206
TAS-20 Total	0.075	−0.255	−0.148	0.322	0.217	0.059	0.239	0.028	−0.205	0.244	−0.139	−0.122
TAS-20 DIF	−0.008	−0.237	−0.118	0.473 *	0.328	−0.102	0.449 *	0.079	−0.231	0.359	−0.260	−0.138
TAS-20 DDF	−0.067	−0.052	−0.151	0.092	0.061	0.274	0.105	0.031	−0.193	0.208	−0.161	−0.136
TAS- 20 EOT	0.278	−0.235	−0.051	−0.015	−0.024	0.060	−0.209	−0.088	0.025	−0.147	0.219	0.037
BPI Intensity	−0.015	−0.096	0.072	0.254	0.251	0.046	0.198	0.037	−0.127	0.238	−0.327	−0.149
BPI Interference	−0.175	−0.117	−0.086	0.425 *	0.296	−0.137	0.285	0.055	−0.214	0.329	−0.577 **	−0.171

* *p* < 0.05; ** *p* < 0.01. Legend: DERS = Difficulties in Emotion Regulation Scale; TAS-20 = Toronto Alexithymia Scale; TAS-20 DIF = Difficulties in Identifying Feelings; TAS-20 DDF = Difficulties in Describing Feelings; TAS-20 EOT = Externally Oriented Thinking; BPI=Brain Pain Inventory; Words = number of words; IDFD = Italian DisFluency Dictionary; IAffN = Negative Affects; IAffP = Positive Affects; IAffS = Sum of Affects; IAffZ = Neutral Affects; IRefD = Italian Reflection Dictionary; ISensD = Italian Sensory Somatic Dictionary; IWRAD = Italian Weighted Referential Activity Dictionary; IWRRL = Italian Weighted Reflection and Reorganization List; Cov = covariation.

## Data Availability

The data that support the findings of this study are available upon reasonable request from the corresponding author.
